# Assessment of soil fertility and potato crop nutrient status in central and eastern highlands of Kenya

**DOI:** 10.1038/s41598-020-64036-x

**Published:** 2020-05-08

**Authors:** James. N. Mugo, Nancy N. Karanja, Charles K. Gachene, Klaus Dittert, Shadrack O. Nyawade, Elmar Schulte-Geldermann

**Affiliations:** 1 The CGIAR Research Program on Roots, Tubers and Bananas (RTB), International Potato Center, Sub-Saharan African Regional Office, P.O BOX 25171-00603, Nairobi, Kenya; 2 Department of Land Resource Management and Agricultural Technology, University of Nairobi, P.O. Box 29053-00625, Nairobi, Kenya; 3 Department of Crop Sciences, Plant Nutrition and Yield Physiology, University of Gottingen, Carl-Sprengel-Weg 1, 37075 Göttingen, Germany; 4 University of Applied Science Bingen (TH Bingen), Berlinstrasse 109, 55411 Bingen, Germany

**Keywords:** Agroecology, Sustainability

## Abstract

Inherent low soil fertility remains a hindrance to potato production in Kenya and continues to pose a threat to food security. A study was conducted in Nyandarua and Meru counties to assess the soil fertility status in smallholder potato farms. Soil and plant tissue samples were collected and analysed for selected nutrients (pH, OC, N, P, K, S, Ca, Mg, Zn, B and Cu) from 198 farms. Critical nutrient levels were used to assess the sufficiency levels of nutrients for potato growth. Soils in the sampled farms were weakly to strongly acidic (pH-CaCl_2_ 3.9–6.6) and had low to high soil organic matter content (1.5–97.5 g Kg^−1^). The percent of farms in Meru and Nyandarua with nutrient contents below critical levels were 66% and 20% for N, 46% and 85% for P, 67% and 31% for S, 9% and 51% for Cu, and 87% and 80% for B, respectively. Low tissue nutrient concentrations were observed for N, P, K, and S irrespective of the sites. Soil pH correlated strongly with majority of the analyzed soil and tissue nutrients. These results affirm the need to design integrative soil fertility management strategies to correct the impoverished soil fertility status in the study area.

## Introduction

The productivity of potato crop (*Solanum tuberosum* L.) in sub-Saharan Africa is greatly constrained by the impoverished soil fertility caused mainly by poor soil nutrient management strategies^1,2^. Potato is a heavy feeder crop with regard to the primary nutrients (N, P and K). For instance, to attain tuber yield of 48 tons ha^−1^, potato tubers remove 47.6 kg N, 24 kg P, 103.4 kg K and 5 kg S, while the haulm requires 31.8 kg N, 8.2 kg P, 47.6 kg K and 3.2 kg S^3^. These nutrient amounts can only be supplied through fertilizer application, a strategy that may be beyond the means of the resource constrained smallholder farmers^4,5^. Nitrogen supply influences tuber bulking rate and the time of tuber growth^6^, K plays an important role in increasing tuber yield, size and quality^7^, while P enhances root development, tuber set and promotes tuber maturity^3^. Sulphur is an integral component in proteins and activates many enzymes regulating potato growth. Soil pH, SOM, Ca, Mg, Fe, Zn, Mn, Mo, Ni, and B are also essential for potato growth and development^3,8^.

In Kenya, productivity of potato averages 8–15 t ha^−1^ which is far much below the potential yield of 40 t ha^−1^ ^9,10^. This yield constraint has been attributed to among other factors, poor nutrient management strategies, poor cropping systems, accelerated soil erosion rates and high cost of inorganic fertilizers^2,8–10^. Fertilizer applications in Kenya is mainly blanket and is often below the recommended rates resulting in inadequate amount of nutrients which cannot meet the potato growth requirement^9,11,12^. Farmers apply mainly di-ammonium phosphate at planting and hardly top-dress with N fertilizer, a practice which has been associated with reduction in soil pH^10,13^. Once the soil pH drops below 4.9, nutrient deficiencies and toxicities become more common. In particular, Mn and Al toxicity and P, K, Ca, and Mg deficiencies^14,15^. The problem may not be prevalent through the entire field but may occur in smaller areas where the soil consists of higher sand or lower organic matter content^16^.

Continuous cultivation of crops without optimal nutrient replenishment has been associated with deficiency of certain nutrients. For instance K which has been known to be adequate in Kenya highland soils has shown depleting levels partly because of high uptake by high K demanding crops such as potato^17,18^. Therefore, to achieve optimum potato yields in Kenya, there is need to supply adequate amounts of both macro and micronutrients in their correct form, quantity and at the right time. Rosen emphasized that imbalances in the supply of nutrients may make certain micro nutrients in the soil unavailable for potato uptake^19^. This reiterates the law of limiting nutrient which states that if one nutrient is limiting, an increase in the yield will be determined by the addition of the same nutrient and thus necessitates the need to identify limiting nutrient^20^.

Addressing the low nutrient use among the smallholder potato growers in Kenya should thus be based on identification of limiting soil nutrients. Soil tests accompanied by plant tissue analysis provides a basis for predicting potential limiting nutrient supply and enable corrective action before serious nutrient deficiencies develop^21,22^. Tissue nutrient analysis is based on the fact that maximum yield and quality of tubers are associated with an optimum range of nutrient in the plant tissue^23^. Nutrient levels falling outside this optimal range are considered growth limiting and require corrective measures^24^. Critical nutrient level is the lower limit of the optimal nutrient range. The use of critical nutrient level for determination of limiting nutrient should be carefully done to take care of the several factors affecting it such as plant part sampled and sample preparation during analysis^25,26^. Once well established, critical nutrient level can be used widely for the same crop^21^. Critical nutrient levels for potatoes at different growth time and plant parts have been described for use in interpretation of plant analysis^27–29^. Soil nutrients sufficiency levels are largely influenced by the soil extraction methods. Interpretation of the soil analysis result in Kenya has generally followed the general recommendations even though there are increasing calibrations of new test methods^30^. The national research and partners have published a manual to guide researchers conduct their activities and interpret their results^24^.

Individual examination of analysed soil chemical element can lead to wrong interpretation of soil chemical property influencing the soil fertility. Thus to determine the key nutrients affecting the nutrient status, there is need for a more robust analytical method. Factor analysis has been used to analyse soil chemical properties in a bid to reduce the factors^31^. It enables the identification of key elements among the many that are analysed.

Fertilizer application if based on key nutrient limitations will allow the growers to adjust nutrient applications according to crops needs, growth rates and length of season. For most accurate fertilizer recommendations, soil test interpretations should be based on local or regional research^19^. Development of site-specific nutrient limit norms for potato will enhance the nutrient use efficiencies and avoid yield losses, and negative environmental impacts by fertilizer use^32,33^. It is in view of this background that this study was conducted with the aim of determining the nutrients status in two major potato producing areas of Kenya. This information is useful for designing integrative nutrient management strategies appropriate to the smallholder farmers.

## Methodology

### Sampling area

**Figure 1:**
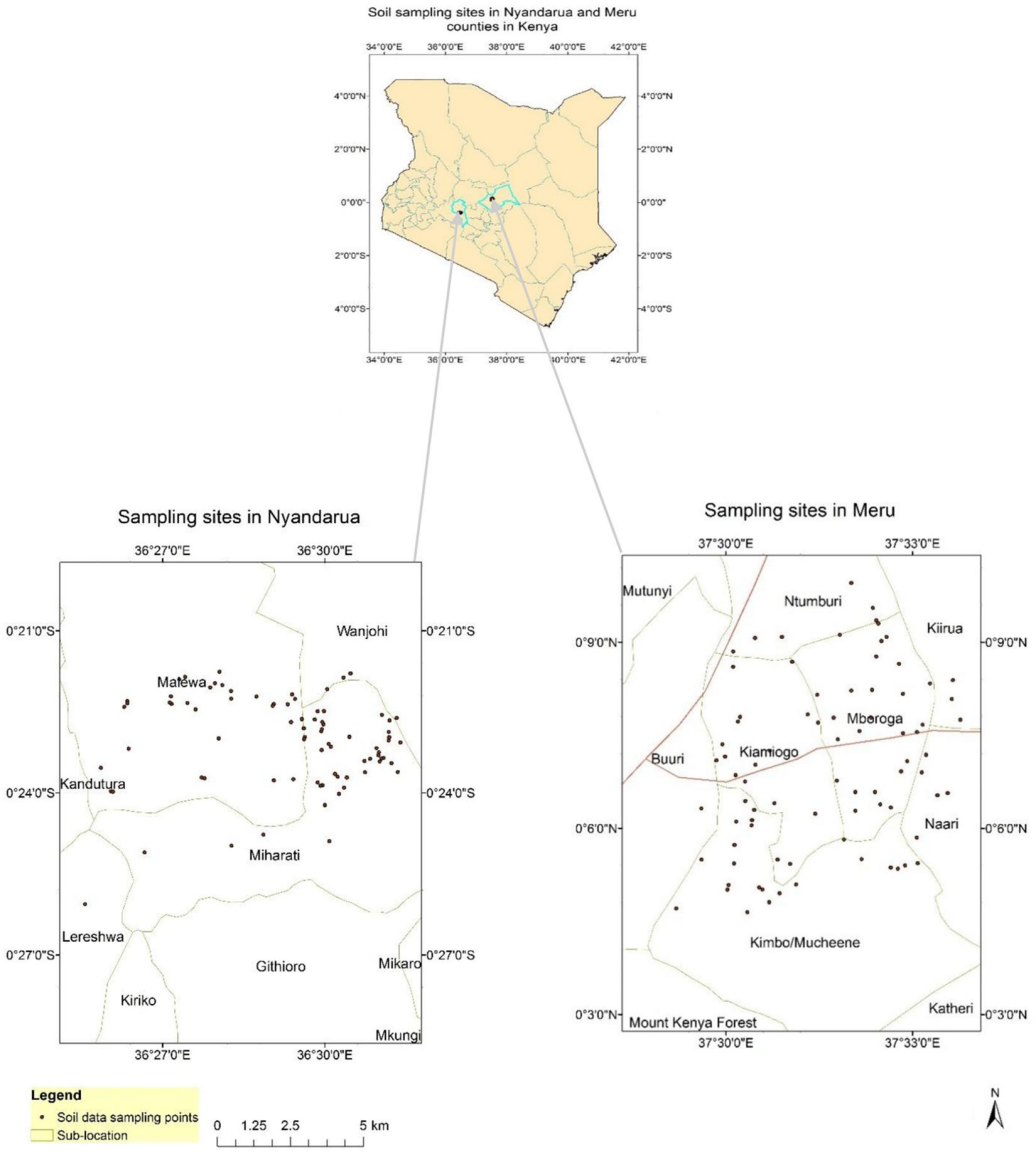
Map of Kenya with sampled counties highlighted and the actual sampled farms in Meru and Nyandarua counties.

This study was conducted in Meru and Nyandarua counties of Kenya (Fig. 1). The two counties are representative of the major potato growing areas of eastern and central Kenya. Sampling in each county was done within a two-kilometer wide transect. The transect in Meru was layed along coordinate range of 0°07′ N, 37°48′ E to 0° 13′ N, 37°53′ E, and along 0°38′ S, 36°50′ E to 0°37′ S, 36°45′ E in Nyandarua. The Meru transect cut across agro-ecological zones; Upper highlands (UH3) (2100–2450 meters above sea level (masl)) and Lower highlands (LH4) (1850–2000 masl) whereas in Nyandarua it covered UH2 (2000–2150 masl), UH3 (2100–2450 masl) and LH3 (1900–2000 masl)^34^. Nitisols is the predominant soils type in Meru while Nyandarua is dominated by Andosols and Planosols^34^. Farms were selected purposively from a list of farmers generated with the assistance of the area agricultural extension officers. The overall sampling targeted farms with potatoes that had attained mid flowering stage as this is the time when peak nutrient uptake by potato occurs. A total of 100 and 98 farms were sampled in Meru and Nyandarua counties, respectively.

### Soil and tissue sampling

Soils were sampled from each target farms in zigzag pattern and at an interval of approximately 10 m. On average, ten (10) soil replicates were taken within rows and inter rows of each farm with a 15 mm diameter soil auger at 0–30 cm depth. The samples were mixed into a composite for each farm and a sub sample of 100 g fresh weight was taken to the laboratory and frozen at 4 ^o^C until analysis. Extraction of soil samples for analysis of Ca, P, K, Mg, B, Zn, and Cu was done using Mehlich 1 procedures^35^ and determined using inductively coupled plasma-optical emission spectrometry (ICP-OES)^36^. Calcium chloride (0.0125 M) was used to extract soil mineral N and S^37^.

Tissue samples were extracted at mid flowering stage from the youngest fully expanded potato leaves (4^th^ leaf from the top of the growing tip). This leaf was considered the critical part for tissue nutrient analyses because younger or older tissue have varying nutrient concentrations which can lead to erroneous interpretations^16^. All leaflets were put into khaki paper bags embedded with silica gel to absorb the moisture. Foliar samples were oven dried at 60 °C, ground using Wiley mill, wet digested, and analysed for mineral nitrogen calorimetrically using Skalar, and for Ca, P, S, K, Mg, B, Zn, S and Cu using ICP-OES after dissolving the ashes (550 °C, 4 h) in dilute HCl^36,38^.

In addition to plant and soil samples, information on the fertilizer type, amounts and manure used was collected using structured questionnaire in order to determine soil nutrient replenishment of the sampled farms.

### Data analysis

Descriptive statistical analyses including means, range and standard deviations were used to explore the data using STATA software and graphs plotted in MS Excel. Nutrient sufficiency ranges (low, adequate and excess) (Table 1) were used to group the farms depending on respective nutrients levels^24,27^. The proposed leaf nutrient sufficiency were adopted from elsewhere since no documented limits exist for potato in Kenya thus are used for general interpretation while critical soil nutrient levels were extracted from a manual published by national research^24,27^. Factor analysis was performed to determine principal soil nutrients elements that influenced the soil fertility status. The data was log transformed for standardization before running factor analysis. Standardization of the data was done since the elements were represented in different units and the concentration varied widely between the various elements. For better interpretation of the results the correlation matrix was rotated. Elements with factor loading of more than 0.5 were considered to be the most influential within a factor. Relationships between soil nutrient content and leaf nutrient concentration, and interrelations of soil nutrients were explored by correlation (r) analysis.

**Table 1:** Soil and tissue nutrient critical levels of macro and micronutrients used in this study.

Element	Soil test	Tissue nutrient concentrations
	Critical level	Critical level
Soil pH (CaCl_2_)	5.5	—
SOC (g kg^−1^)	25.0	—
N (g kg^−1^)	2.5	44.0
P (mg kg^−1^)	30.0	2.5
K (Cmol kg^−1^)	0.2	39.0
S (mg kg^−1^)	4.5	3.0
Ca (Cmol kg^−1^)	0.9	9.0
Mg (Cmol kg^−1^)	0.3	2.5
Zn (mg kg^−1^)	0.6	19.0
B (mg kg^−1^)	1.0	24.0
Cu (mg kg^−1^)	0.2	5.0

Source: Soil critical levels^24^, Tissue critical levels^27^.

## Results

### Fertilizer and manure use

**Figure 2:**
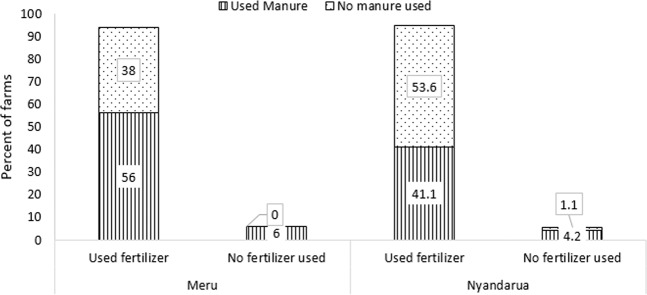
Percent of farms in which mineral fertilizer and/or cattle manure was used in Meru and Nyandarua.

**Figure 3:**
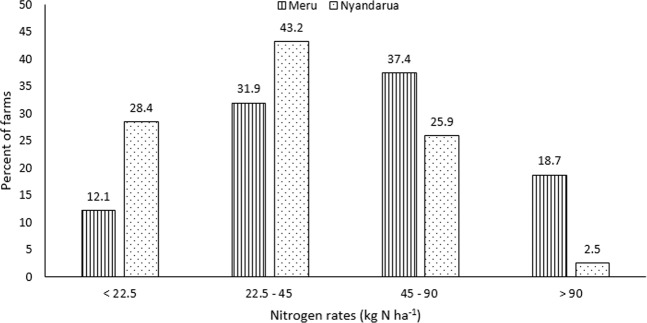
Percent of farms in which fertilizer was applied at various rate of nitrogen per hectare in Meru and Nyandarua.

Fertilizer was applied in majority of the sampled potato farms in Meru (94%) and Nyandarua (95%) (Fig. 2). This fertilizer was mainly in the form of di-ammonium phosphate. Manure was applied in 62% and 45% of sampled farm in Meru and Nyandarua respectively. Combined application of fertilizer and manure was done in 56% and 41% of sampled farms in Meru and Nyandarua respectively. With respect to N, which is considered the major limiting nutrient in Kenya, the majority of the farmers applied lower rates of this nutrient than the recommended rate of 90 Kg N ha^−1^ for these areas (Fig. 3). Generally, majority of the farmers in Meru applied N at rates greater than the recommended compared to farmers in Nyandarua.

### Soil chemical characteristics and tissue nutrient content

In Nyandarua, the soil pH (CaCl_2_) ranged from 3.9 to 6.2, with an overall mean of 5.0 ± 0.5, with 86% of sampled farms having values below the critical level of 5.5 (Table 2). Similar results were observed in Meru which showed a soil pH range of 4.2–6.6 with 55% of the sampled farms showing pH values below the critical levels for potato production. The SOC contents ranged between 1.4–91.5 g kg^−1^ in Meru and 19.7–65.2 g kg^−1^ in Nyandarua, and were below the critical levels for potato growth in 9% of sampled farms in Nyandarua and 30% of sampled farms in Meru. Total N varied widely between the sampled farms, both in Meru and Nyandarua, with 67% and 20% of the sampled farms recording values below critical levels, respectively. Soil P showed the highest variations between the sampled farms in Meru and Nyandarua with 47% and 85% of the sampled farms having this element at concentrations below the critical levels. Sulphur was below critical levels in 68% and 32% of sampled farms in Meru and Nyandarua respectively. Over 80% of the sampled farms in both regions had B below the critical levels. Zinc content showed similarity in Meru (5.1 ± 3.5) and Nyandarua (5.6 ± 5.3). Calcium and Mg were generally optimal in Meru and Nyandarua. Ca-Mg ratio was higher in Meru (3.63 ± 0.88) compared to Nyandarua (1.98 ± 0.77).

**Table 2:** Summary statistics (Mean, SD, Minimum and Maximum) and percent of farms below critical levels for potato growth in Meru and Nyandarua regions.

Variable	Meru (N = 99)					Nyandarua (N = 93)				
	Min	Max	Mean	Std. Dev.	% farms below critical value	Min	Max	Mean	Std. Dev.	% farms below critical value
pH (CaCl_2_)	4.2	6.6	5.4	0.6	54.5	3.9	6.2	5.0	0.5	86.2
SOC (g kg^−1^)	1.4	91.5	31.3	13.6	30.3	19.7	65.2	37.2	10.4	8.6
N (g kg^−1^)	<0.1	9.0	2.8	1.4	66.7	1.8	5.9	3.3	0.9	20.2
P (mg kg^−1^)	3.4	258.8	47.1	44.2	46.5	0.9	430.2	23.7	54.5	85.1
K (Cmol kg^−1^)	0.2	2.7	0.9	0.5	—	0.1	1.6	0.5	0.4	18.1
S (mg kg^−1^)	1.1	14.1	4.1	2.4	67.7	1.9	20.8	6.1	3.0	31.9
Ca (Cmol kg^−1^)	1.3	12.3	4.6	2.2	—	1.4	11.4	4.4	2.0	—
Mg (Cmol kg^−1^)	1.0	5.4	2.4	0.9	—	0.4	2.9	1.3	0.5	—
Zn (mg kg^−1^)	1.0	20.1	5.1	3.5	—	0.6	27.5	5.6	5.3	—
B (mg kg^−1^)	0.1	2.3	0.7	0.3	87.9	<0.1	2.6	0.7	0.4	80.9
Cu (mg kg^−1^)	<0.1	4.3	1.4	1.1	9.1	<0.1	1.7	0.3	0.2	51.0
C-N ratio	8.9	13.3	11.2	0.7		8.7	13.3	11.5	0.8	
Ca-Mg ratio	0.8	4.8	2.0	0.8		1.0	6.2	3.6	0.9	

Tissue N concentrations exhibited wide variations between the sampled farms with a mean of 40.27 ± 5.32 g kg^−1^ in Meru and 47.39 ± 4.98 g kg^−1^ in Nyandarua (Table 3). About 73% of the sampled farms had N below optimal levels in Meru compared to 23% in Nyandarua. Potassium variability was similarly high between the sampled farms averaging 47.33 ± 10.92 g kg^−1^ in Meru and 51.64 ± 12.52 g kg^−1^ in Nyandarua. The sampled farms with K levels below the optimal levels were 22% and 15% respectively in Meru and Nyandarua. Zinc concentrations varied between 18.3–78.9 mg kg^−1^ and 22.5–101.8 mg kg^−1^ which is within the optimal levels for potato growth. Boron and Cu similarly showed wide variations between the sampled farms averaging 31.45 ± 3.08 mg kg^−1^ and 23.70 ± 8.99 mg kg^−1^, and 9.79 ± 3.08 and 11.62 ± 4.46 mg kg^−1^ respectively in Meru and Nyandarua. The sampled farms with B levels below critical limits were 17% and 55% respectively in Meru and Nyandarua.

**Table 3:** Summary statistics (mean, SD, Minimum and Maximum) and percent of farms below critical levels of plant nutrient content in Meru and Nyandarua regions.

Plant nutrients	Meru (N = 99)					Nyandarua (N = 93)				
	Min	Max	Mean	Std. Dev.	% farms below critical value	Min	Max	Mean	Std. Dev.	% farms below critical value
N (g kg^−1^)	25.9	56.3	40.3	5.3	73.0	33.3	60.6	47.4	5.0	22.7
P (g kg^−1^)	1.9	5.3	3.2	0.8	24.0	2.4	5.8	3.7	0.7	1.2
K (g kg^−1^)	16.7	73.2	47.3	10.9	22.0	14.8	76.1	51.6	12.5	14.9
S (g kg^−1^)	2.7	5.3	3.5	0.5	18.0	2.8	12.4	4.5	1.3	1.2
Ca (g kg^−1^)	6.5	27.1	15.5	3.3	0.0	12.7	36.4	18.2	4.2	0.0
Mg (g kg^−1^)	5.0	16.6	9.0	2.6	0.0	4.4	16.6	7.8	2.3	0.0
Zn (mg kg^−1^)	18.3	78.9	31.9	9.4	2.0	22.5	101.8	37.7	10.6	0.0
B (mg kg^−1^)	15.8	44.7	31.5	6.5	17.0	6.5	42.1	23.7	9.0	55.2
Cu (mg kg^−1^)	3.5	16.3	9.8	3.1	5.0	5.0	26.0	11.6	4.5	1.2

### Relationship between soil chemical properties and plant nutrient content

Tissue phosphorus was significantly positively correlated to pH (r = 0.37), soil P and negatively correlated to soil Cu (r = −0.40) (Table 4). A significant positive correlation between tissue Ca and soil N (r = 0.49) and tissue and soil Ca (r = 0.49) was observed. Similarly, tissue K concentrations and soil K contents (r = 0.32) correlated positively. Soil Cu concentrations correlated significantly and negatively with tissue P (r = −0.40) and N (r = −0.40).

**Table 4:** Correlations coefficients (r) between soil pH, soil chemical properties and plant tissue nutrients content in Meru region.

Tissue nutrient	**S**oil nutrients									
	Soil pH	N	P	K	Ca	S	Mg	Zn	B	Cu
N	−0.02	0.22*	0.10	0.03	0.08	0.19	−0.07	−0.05	0.08	−0.08
P	0.37*	−0.01	0.49*	0.25*	0.31*	0.02	0.20*	0.04	0.18	−0.40*
K	0.20*	−0.27*	0.22*	0.32*	0.01	−0.23*	0.05	0.08	0.01	−0.12
Ca	0.27*	0.49*	0.13	0.01	0.49*	0.33*	0.07	0.08	0.34*	−0.40*
S	0.10	−0.14	0.11	0.01	−0.02	−0.10	0.13	−0.13	−0.07	−0.13
Mg	−0.21*	0.20*	−0.23*	−0.31*	−0.06	0.29*	−0.01	−0.15	0.03	0.30*
Zn	−0.09	−0.06	−0.08	−0.09	−0.11	0.01	−0.12	0.09	−0.03	0.06
B	−0.21*	−0.39*	−0.22*	−0.05	−0.36*	−0.06	−0.25*	0.06	−0.23*	0.26*
Cu	−0.30*	−0.59*	−0.24*	−0.18	−0.52*	−0.19	−0.09	−0.41*	−0.40*	0.44*

*level of significant P < 0.05.

In Nyandarua, N uptake correlated negatively with concentration of other nutrients in the soil (Table 5). The concentration of tissue K positively correlated with the concentration of soil P (r = 0.25), Ca (r = 0.22), Mg (r = 0.27) and B (r = 0.25). The tissue concentration of Cu was negatively correlated with the soil pH (r = −0.24) and concentration of N (r = −0.37), P (r = −0.3), Ca (r = −0.3), S (−0.33) and B (r = −0.37). Increase in soil pH and concentration of P, K and Zn increased the uptake of Zn by potato crop in Nyandarua.

**Table 5:** Correlations coefficients (r) between pH, soil chemical properties and plant tissue nutrients content in Nyandarua region.

Tissue nutrient	Soil nutrients									
	Soil pH	N	P	K	Ca	S	Mg	Zn	B	Cu
N	−0.46*	−0.11	−0.22*	−0.38*	−0.26*	−0.07	−0.1	−0.18	−0.28*	0.29*
P	0.13	−0.18	0.41*	0.25*	0.16	−0.07	0.05	0.10	0.02	−0.09
K	0.40*	0.09	0.25*	0.57*	0.22*	0.16	0.27*	0.21	0.25*	−0.14
Ca	0.13	0.26*	0.12	−0.15	0.21	0.11	0.05	0.16	0.23*	−0.17
S	0.11	0.11	−0.03	0.07	0.04	−0.01	0.05	0.24*	0.02	−0.14
Mg	−0.30*	0.24*	−0.17	−0.49*	−0.1	−0.11	0.07	0.04	−0.07	0.12
Zn	−0.07	0.03	−0.22*	0.07	−0.1	−0.12	−0.04	−0.07	−0.09	0.04
B	0.34*	0.10	0.24*	0.25*	0.21	0.08	0.10	0.22*	0.19	−0.24*
Cu	−0.24*	−0.37*	−0.30*	−0.16	−0.30*	−0.33*	−0.18	−0.20	−0.37*	0.29*

*Level of significant P < 0.05.

### Interrelations of soil chemical properties

Soil pH positively correlated to soil P (r = 0.49), K (r = 0.47), Ca (r = 0.79), Mg (r = 0.69) and B (r = 0.74) and negatively to Cu (r = −0.59) in Meru (Table 6). Copper was also significantly negatively correlated to N (r = −0.44), P (r = −0.39), Ca (r = −0.68), Mg (r = −0.38) and B (r = −0.37). The Ca levels were positively correlated to N (r = 0.63), P (r = 0.51), Mg (r = 0.61), Zn (r = 0.35) and B (r = 0.66). Sulphur was not significantly correlated to other soil chemical properties in Meru while Zn was only significantly correlated to Ca.

**Table 6:** Correlations coefficients (r) of soil chemical properties in Meru and Nyandarua region.

Region		pH	N	P	K	Ca	S	Mg	Zn	B
Meru	N	0.31								
	P	0.49*	0.14							
	K	0.47*	0.18	0.36*						
	Ca	0.79*	0.63*	0.51*	0.27					
	S	−0.06	0.24	−0.06	0.10	0.10				
	Mg	0.69*	0.18	0.26	0.16	0.61*	−0.19			
	Zn	0.31	0.20	0.28	0.31	0.35*	−0.02	0.18		
	B	0.74*	0.49*	0.29	0.57*	0.66*	0.17	0.41*	0.31	
	Cu	−0.59*	−0.44*	−0.39*	−0.25	−0.68*	−0−0.08	−0.38*	−0−0.22	−0.37*
Nyandarua	N	0.35*								
	P	0.46*	0.28							
	K	0.70*	0.27	0.54*						
	Ca	0.77*	0.49*	0.47*	0.54*					
	S	0.33	0.28	0.36*	0.31	0.29				
	Mg	0.57*	0.55*	0.27	0.49*	0.76*	0.10			
	Zn	0.28	0.40*	0.27	0.25	0.43*	0.29	0.34*		
	B	0.75*	0.56*	0.45*	0.64*	0.77*	0.38*	0.58*	0.43*	
	Cu	−0.46*	−0.17	−0.20	−0.37*	−0.41*	−0.31	−0.22	−0.25	−0.42*

*Level of significant P < 0.05.

The correlation of soil pH to other soil chemical properties in Nyandarua was positive for N (0.35), P (0.46), K (0.7), Ca (0.77), Mg (0.57) and B (0.75). Similarly, Cu was negatively correlated to all the nutrients and significantly with pH (−0.46), K (−0.37), Ca (−0.41) and B (−0.42). Boron was positively correlated to all analysed nutrients apart from the correlation with Cu which was negative.

### Principal soil nutrients influencing soil fertility

Factor analysis for soil chemical properties in Meru retained 7 principal factors with the first three factors accounting for 91% proportion of the variance. Zinc and S were the most unique soil chemical properties in Meru (Table 7). The first factor was mainly weighted by soil pH, Ca, Mg and B whereas the second one was due to N and SOC. The Third factor was influenced by K and B. Soil Cu had a negative influence on the first factor (soil pH). Factor analysis results for the soil chemical properties in Nyandarua showed that the first three factors explained 90% of the variance. All the soil chemical properties (except Cu) which had major influence on the first factor in Meru had greater influence in the first factor in Nyandarua as well. The second factor in Nyandarua was dominated by SOC and N. Zinc and S were unique chemical properties in Nyandarua. In addition, Cu was found to be unique in Nyandarua.

**Table 7:** Factor loading for the first 3 factors and unique variances of soil chemical properties in Meru and Nyandarua.

Variable	Meru				Nyandarua			
	Factor 1	Factor 2	Factor 3	Uniqueness	Factor 1	Factor 2	Factor 3	Uniqueness
pH	**0.88**	0.15	0.32	0.09	**0.80**	0.15	0.22	0.21
N	0.17	**0.98**	0.07	0.01	0.20	**0.94**	0.15	0.03
C	0.15	**0.98**	0.08	0.01	0.19	**0.97**	−0.03	0.02
P	0.41	0.05	0.21	**0.52**	0.38	0.08	**0.69**	0.37
K	0.23	0.09	**0.63**	**0.51**	**0.62**	0.11	0.40	0.33
Ca	**0.77**	**0.50**	0.06	0.07	**0.85**	0.29	0.17	0.13
S	−0.13	0.27	0.15	**0.66**	0.17	0.19	0.32	**0.64**
Mg	**0.76**	0.06	−0.03	0.37	**0.72**	0.47	0.00	0.17
Zn	0.22	0.15	0.28	**0.77**	0.31	0.30	0.16	**0.65**
B	**0.56**	0.36	**0.57**	0.21	**0.68**	0.38	0.25	0.24
Cu	−0**.53**	−0.35	0.01	0.42	−0.41	−0.04	−0.06	**0.66**

## Discussion

The differences in soil chemical properties between Meru and Nyandarua were due to the differences in soil types and fertilizer and manure use. The farms examined in Meru were mainly dominated by Nitisols, which are acidic with soil organic matter content ranging from low to high^39^. Soils in the sampled farms in Nyandarua were dominated by Planosols which are formed from volcanic ash and are regarded as degraded with the surface soil being acidic^40^. Planosols due to their imperfect drainage and waterlogging, have poor soil organic carbon build-up capacity compared to the well-drained Nitisols in Meru. Organically bound nutrients, especially N and P are not available to plants because it cannot be absorbed into root cells without first being released from the organic molecule through mineralization^41^. This process is regulated by soil micro-organisms that work at optimal aeration that is not effectively provided in Planosols. Differences in soil nutrient levels and therefore tissue nutrient concentrations between Meru and Nyandarua suggest differences in cropping systems. Where the previous cropping system has caused a depletion of soil organic matter, the soils are more likely to be acidic with limited capacity to hold N, P, K, Ca and some essential micronutrients. This coupled with the low fertilizer and manure use, meant increased soil degradation. Long term fertilization regime and manure use affects nutrient concentration, SOM and microbial life in the soil^42^. It can therefore be concluded that the farms in the two study areas had a slight nutrient differences.

The low soil pH in both Meru and Nyandarua relates to the high rainfall amounts that probably caused cation leaching. Oxidation of DAP fertilizer commonly used by the farmers in these areas results in the formation of strong inorganic acids such as nitric acid which further lower the soil pH^10^. The volcanic parent material also adds up to the low acidity as the two soil types in the study area are described as acidic soils^39^. Low soil pH in turn affects the uptake of most macro and secondary plant nutrients by either its effects on microbial activity and dissolution of Al/Fe ions. Nitrogen, K and S are less affected by pH but P is greatly affected, while micro nutrients are mostly available in slightly acidic soil^43^.

The relatively low soil N content in the sampled farms in Meru was reflected in the tissue N levels and could be attributed to the low soil pH and coupled with the low soil organic carbon contents. Soil organic matter retains soil N and prevents it from leaching beyond the active rooting zones^3,44^. Microbial activity that releases N from organic matter and certain fertilizers are particularly affected by the low soil pH since microbial activities occurs best at soil pH range of 5.5 to 7.0^45^. In a study conducted to compare the yield response of potatoes to increasing levels of N, a larger response was exhibited in soils with higher soil organic matter content irrespective of the amount of N applied, and the recovery of the applied N was greater where the soil pH was slightly above 6.0^46^. The large number of farms with N nutrient below optimum levels could thus be as a result of nutrient leaching, low amount of N replenishment and high nutrient mining as a result of continuous farming^3,47^. According to Westerman^16^, potato takes up to 235 kg N ha^−1^, which is mined out of the soil in a growing season.

The low soil P content was reflected in tissue P content suggesting reduced uptake. This observation could be ascribed to the predominating clay soils in these study areas^39^ as well as to the low soil pH. Both Nitisol and Planosols examined in this study have clay content >30%^39^. Clay soils and acidic soils have high Al and Fe contents which besides fixing the available soil P are associated with increased soil acidity, thus leading to inconsistent response to soil P by potato leaf uptake^48,49^. The observed low soil phosphorus could be associated with the leaching of P, especially when the soil’s sorption capacity is saturated^50^. There is recent evidence that higher P concentrations are found in the soil water moving in the bypass flow pores within the agricultural farms^51,52^. Poor P recovery through fertilizer application under acidic conditions is due to the fact that the P applied in the form of fertilizers is mainly adsorbed by the soil^48^. Furthermore, P is largely transported offsite attached to the sediment to be later released via dissolution or made available when anoxic conditions are present^13^. The reduction of transport would also mean reduced uptake.

Soil K in Meru was adequate however this was not the case with the tissue K concertation. The differences between soil K and its uptake in Meru could be as a result of cation balances. A study has shown Mg induced K deficiency on Nitisol^53,54^. Soil moisture also affects the uptake of K since it helps in mass flow movement^55^. Lower soil moisture in Nitisol would thus mean reduced K uptake. Further to this, low soil pH indirectly affects K uptake. At low soil pH, Al becomes soluble thus dominating CEC hence lowering the soil capacity to hold K^56^. Potassium is of high importance in potato growing as it affects tuber yield and qualities and it is the highest absorbed nutrient^16^.

Leaf S concentration was appreciably lower than the critical concentration. This was an indication that S deficiency is a problem in the potato growing farms in Meru and Nyandarua. This observation is consistent with other findings that have established that potatoes do not respond to sulphur applications except in extremely deficient soils^57,58^. If soil test S is less than 7 ppm and/or tissue S is less than 0.18%, then 20 kg S per hectare should be banded at planting^8,27,59^. Factors contributing to this increased incidence of S deficiency among the examined farms may be related to the use of S-free fertilizers such as urea and di-ammonium phosphate^9,60^. This is despite the fact that potato tubers remove a high amount of S; typically 1 tonne of potato tubers will remove 4.5 kg of S^8^. The low soil organic matter could also lead to low S as 95% of S is known to be associated with organic matter^8^.

Boron and Cu were generally limiting in the soils tested, an observation which was reflected in the potato tissues. This observation could be asserted to the limited supply of these nutrients in the soil and to the overall low soil organic matter contents of the sampled soils^61^. Low soil pH would also be a contributing factor as a result of Al solubility that interferes with uptake and transport of other nutrients^8^. The high level of B deficiency in the sampled farms in Meru could be associated with nutrient leaching coupled with low soil pH which hinders B uptake by potato^62^. Elsewhere, B deficiency has been related to the low soil organic matter contents, especially under prevailing cold wet weather and in periods of drought^8,61^.

The significant positive correlation between leaf P and soil P relates to the low P ions in the soil solution at the root surface. This is in accordance with Morgan & Connolly^63^ who observed that potato responds to nutrient deficiency by changing its root structure so as to increase the overall nutrient acquisition. Burke^8^ similarly argued that the ability of potato crop to absorb P will depend on the concentration of P ions in the soil solution at the root surface. The significant interaction between tissue P concentrations and soil P contents indicates that this element was limiting in majority of the sampled farms. Soil pH and tissue P content similarly showed significant associations implying their interactive effects. These relations could be greatly attributed to the low content of active P forms in the highly weathered clay dominating soils in this study sites^39^, since in acidic soils, P may be adsorbed by Fe or Al oxides, and various clay minerals^3^.

The significant relationship between tissue K concentrations and soil K contents reflects soil K deficiency in several of the sampled farms. Though the tropical soils are generally considered to be sufficient in K, deficiency of this element was evident in this study. Potatoes take up more K than many other arable crops^16,64^. During peak vegetative growth, potatoes may require 10 kg K_2_O ha^−1^ per day from the soil^65^. At about 80 days after emergence high yielding potato crop may remove more than 500 kg K_2_O ha^−1^ ^8^. At harvest, more than 75% of the K uptake is found in the tubers, which typically contain around 5.8 kg K_2_O per tonne of tubers^8^. As the potato crop is harvested and the tubers are removed from the field, so K is taken away in that crop material. This must be replaced otherwise future crops will be grown in soil with a reduced K level, resulting in low yields.

Significant negative relations were exhibited between tissue Cu nutrient content and soil N, P, Ca, B, and Zn indicating that tissue Cu concentrations seems to level off at certain concentrations of these elements. Similar results have been reported elsewhere^66^. This would suggest that Cu toxicity is becoming a greater concern in Nitisols and Planosols. The significant correlation between tissue Cu concentrations and soil Cu content however indicated that Cu was a limiting nutrient requiring corrective measures. The positive association between tissue magnesium and soil Cu content showed a synergy between these two nutrient elements. This would suggest that Mg uptake by potato increases with increasing level of Cu concentrations in the soil. The positive correlation between tissue Ca and soil N, S, and B would mean Ca uptake by potato is more efficient in soils with optimum N, S, Ca and B levels. The role of Ca and B on cell wall formation and auxin transport thus explains the correlation^67^.

Soil pH is the single most factor affecting soil nutrients due to its effects on microbial activity and nutrients dissolution^8^. High concentrations of Ca^2+^ in the soil solution increases the soil pH which reduces the solubility of Al and Fe ions and enhances solubility and availability of certain micro-nutrient elements. Boron levels are associated with soil organic matter thus the low soil organic matter could be the reason for B correlations with other soil nutrients. This implies that the trend of soil nutrient elements depletion is similar and that with continuous mining, other nutrients will become limiting. The negative effect of Cu levels to most of other soil nutrients has been reported in other studies^68,69^. Copper has a negative effect on soil bacteria which would in turn affect other soil nutrients concentrations^70^.

Factor analysis results showed that soil pH and CEC related to soil chemical properties (K, Ca, Mg) influenced the first factor. The higher the bases (K, Ca, Mg) the higher the soil pH. This would thus reinforce that soil pH is a major factor to consider for sustainable nutrient management^61^. The second factor affecting soil fertility in the two regions was soil organic matter. Soil organic matter retains nutrients especially N, P and S. Soil organic matter is subjected to change by cropping system and management practices and its influence on soil fertility is thus important^8,61^. Management of the soil pH and soil organic matter factors would thus improve soil fertility in the studied region.

The low soil nutrient contents measured for N, P, K, S and B were reflected in the tissue nutrient concentrations which were consistently lower indicating that nutrient uptake by potato crop was influenced by soil nutrient concentrations. The correlations were however not very strong which could be explained by other confounding factors influencing nutrient uptake such as crop variety, farm management, climatic factors and crop health status^16,71,72^. Similar relations have elsewhere been reported^73,74^. Nutrient interaction in the soil and within the plant also affects crop nutrient uptake^66,75^.

## Conclusions

This study shows that N, P, K, S and B are the key limiting nutrients to potato production in the major potato growing areas of Kenya. These nutrients need to be integrated in nutrient management programs for major potato growing areas of Kenya. Soil pH was found to be low in all the sampled farms and was the major factor that influenced nutrient uptake by potato crop. Improved cropping systems and soil management practices are required to adjust the soil pH for enhanced soil fertility in the study areas.

## Data Availability

Data is available from corresponding author.
